# Dr. William H. Jackson

**Published:** 1904-01

**Authors:** 


Dr. William H. Jackson, of Springville, N. Y., died at his home November 28, 1903, aged 62 years. It is reported that Dr. Jackson's health had been impaired for some time, but that pneumonia was the immediate cause of his death. He was born at Clarkson, N. Y., August 26, 1841, received his preliminary education at the Albany State Normal School and took his doctorate degree from the University of South Carolina at Columbia in 1873. Dr. Jackson occupied a position of prominence not only in his profession but in the community where he lived. He was a member of various medical societies and of masonic and other orders. In 1863 Dr. Jackson married Miss Mary Hyde. Her death occurred in 1870. In 1877 Dr. Jackson married Frances Rockwell. From the first union three children survive, Mrs. C. R. Shuttleworth, of Buffalo; Dr. W. H. Jackson, of Galeton, Pa., and Lucian P. Jackson, of Warren, Pa. Besides his widow, two sons, by the last marriage, also survive. They are Fletcher R., and Lawrence C. Jackson.

